# Trajectories of Inflammation in Youth and Risk of Mental and Cardiometabolic Disorders in Adulthood

**DOI:** 10.1001/jamapsychiatry.2024.2193

**Published:** 2024-08-21

**Authors:** Edward R. Palmer, Isabel Morales-Muñoz, Benjamin I. Perry, Steven Marwaha, Ella Warwick, Jack C. Rogers, Rachel Upthegrove

**Affiliations:** 1 Institute for Mental Health, School of Psychology, University of Birmingham, Birmingham, United Kingdom; 2 Early Intervention Service, Birmingham Women’s and Children’s NHS Trust, Birmingham, United Kingdom; 3 Department of Psychiatry, University of Cambridge, Cambridge, United Kingdom; 4 Cambridgeshire and Peterborough NHS Foundation Trust, Cambridge, United Kingdom; 5 The Barberry National Centre for Mental Health, Birmingham and Solihull Mental Health NHS Trust, Birmingham, United Kingdom

## Abstract

**Importance:**

Research suggests that low-grade, nonresolving inflammation may predate adult mental and physical illness. However, evidence to date is largely cross-sectional or focuses on single disorder outcomes.

**Objectives:**

To examine trajectories of inflammation as measured by C-reactive protein (CRP) levels in a large sample of children and adolescents, and to explore associations between different identified trajectories and mental and related cardiometabolic health outcomes in early adulthood.

**Design, Setting, and Participants:**

In a longitudinal cohort study using data from the large UK-based Avon Longitudinal Study of Parents and Children (ALSPAC), latent class growth analysis (LCGA) was used to explore different trajectories of inflammation, with logistic regression exploring association with mental and physical health outcomes. Participants with measurable CRP data and associated mental and cardiometabolic health outcomes recorded were included in the analysis. Data analysis was performed from May 1, 2023, to March 30, 2024.

**Exposures:**

Inflammation was assessed via CRP levels at ages 9, 15, and 17 years. LCGA was used to identify different trajectories of inflammation.

**Main Outcomes and Measures:**

Outcomes assessed at age 24 years included psychotic disorders, depressive disorders, anxiety disorders, hypomania, and, as a measure of insulin resistance, Homeostasis Model Assessment (HOMA2) score.

**Results:**

A total of 6556 participants (3303 [50.4%] female) were included. Three classes of inflammation were identified: persistently low CRP levels (reference class, n = 6109); persistently raised CRP levels, peaking at age 9 years (early peak, n = 197); and persistently raised CRP levels, peaking at age 17 years (late peak, n = 250). Participants in the early peak group were associated with a higher risk of psychotic disorder (odds ratio [OR], 4.60; 95% CI, 1.81-11.70; *P* = .008), a higher risk of severe depression (OR, 4.37; 95% CI, 1.64-11.63; *P* = .02), and higher HOMA2 scores (β = 0.05; 95% CI, 0.01-0.62, *P* = .04) compared with participants with persistently low CRP. The late peak group was not associated with any outcomes at age 24 years.

**Conclusions and Relevance:**

Low-grade systemic inflammation peaking in midchildhood was associated with specific mental and cardiometabolic disorders in young adulthood. These findings suggest that low-grade persistent inflammation in early life may be an important shared common factor for mental-physical comorbidity and so could be relevant to future efforts of patient stratification and risk profiling.

## Key Points

**Question:**

Are differing trajectories of low-grade inflammation throughout childhood and adolescence associated with an increased risk of developing certain mental and related cardiometabolic health conditions in early adulthood?

**Findings:**

This longitudinal cohort study found that having persistently raised levels of inflammation as measured by C-reactive protein throughout childhood and adolescence, peaking at age 9 years, was associated with an increased risk of developing psychosis disorder, severe depression, and higher levels of insulin resistance.

**Meaning:**

Increased inflammation in childhood may be an important predisposing risk factor to the development of both mental and cardiometabolic disorders in early adulthood.

## Teaser

This longitudinal cohort study using data from the Avon Longitudinal Study of Parents and Children (ALSPAC) examines trajectories of inflammation in childhood and their associations with mental and related cardiometabolic health outcomes in early adulthood.

## Introduction

Mental health and cardiometabolic disorders are the leading sources of health burden globally.^1^ Current management has significant limitations, demanding research, better mechanistic understanding, and ultimately more effective treatments. Inflammation and changes in the immune system have been associated with both mental health and cardiometabolic disorders, offering potential new targets for further research, treatments, and risk profiling.

There is a wealth of evidence triangulated from various types of research linking inflammation with mental disorders. There are strong meta-analytical, cross-sectional data showing increased inflammation in those with depression,^2^ bipolar affective disorder,^3^ and psychosis-spectrum disorders.^4^ Recent large cross-sectional studies indicate an association between proinflammatory states and anxiety disorders.^5^

Longitudinal data exist for both depression and psychosis. A longitudinal study using Avon Longitudinal Study of Parents and Children (ALSPAC) data found that higher interleukin 6 levels at age 9 years were associated with subsequent higher risks of depression and psychotic episodes at ages 18 years^6^ and 24 years.^7^ A meta-analysis has shown that an increased C-reactive protein (CRP) level (>0.3 mg/dL; to convert to milligrams per liter, multiply by 10) is associated with increased rates of psychosis.^8^ However, these studies only examined inflammation at a single time point. One study looked at longitudinal inflammation trajectories as measured by CRP across childhood and found an association between increasing CRP and depression at age 18 years.^9^ Finally, mendelian randomization studies have found a potentially causal effect of genetically predicted levels of inflammatory markers on the risk of depression^10^ and schizophrenia.^11^

Poor cardiometabolic outcomes have long been observed in individuals with mental health disorders, with rates of obesity, type 2 diabetes, and cardiovascular disease being 1.4 to 2 times higher than in the general population.^12^ While the impacts of chronic disease, such as lifestyle and medication, partly explain this, they alone do not appear to explain the entire association. For example, insulin resistance (IR) has been found at higher rates than expected in antipsychotic-naive young patients with first-episode psychosis before the lifestyle changes of disease chronicity.^13^ Similar to mental health disorders, meta-analytical, cross-sectional data^14^ and longitudinal data^15^ show an association of inflammation, including increased CRP levels, with IR and type 2 diabetes. Further, a mendelian randomization analysis suggested that inflammation could be a potential common cause for schizophrenia and IR, which may explain why cardiometabolic traits and schizophrenia commonly co-occur.^11^

Therefore, evidence supports the concept that inflammation in early life may be associated with the risk of developing mental health and cardiometabolic disorders in adulthood. However, previous research has mostly focused on single time points, potentially overlooking dynamic temporal changes in these markers, or single outcomes, potentially overlooking the effect the same inflammatory exposure can have on multiple outcomes. Here, we aim to examine the longitudinal association of inflammation through childhood and adolescence and whether the same classes of inflammation predispose to higher risks of mental and cardiometabolic disorders. Our a priori hypothesis, based on previous research, was that there would be a group of children and adolescents with chronic low-grade inflammation and that this group would be at increased risk of developing mental health and cardiometabolic disorders.^11^ We hypothesized that the strongest association would exist for the conditions for which there is consistent evidence for the role of inflammation, namely psychosis,^6,7,8^ depression,^6,7^ and IR.^15^

## Methods

### Participants

The ALSPAC is a UK birth cohort study examining the determinants of development, health, and disease during childhood and beyond.^16,17^ Pregnant women residing in Avon County, England, with expected dates of delivery between April 1, 1991, and December 31, 1992, were invited to take part in the study. The initial number of pregnancies enrolled was 14 541. With further recruitment at 7 years, the total sample size for analyses using any data collected after the age of 7 years was therefore 15 447 pregnancies.^16^ Study data were collected and managed using REDCap (Research Electronic Data Capture; Vanderbilt) electronic data capture tools hosted at the University of Bristol. REDCap is a secure, web-based software platform designed to support data capture for research studies.^18^ For further details, see the eMethods in Supplement 1. The study website contains details of all the data available through a fully searchable data dictionary and variable search tool.^19^ All participants provided written informed consent, and ethical approval for the study was obtained from the ALSPAC Law and Ethics Committee and relevant local research ethics committees. Informed consent for the use of data collected via questionnaires and clinics was obtained from participants following the recommendations of the ALSPAC Ethics and Law Committee at the time. The study followed the Strengthening the Reporting of Observational Studies in Epidemiology (STROBE) reporting guideline. Data analysis was performed from May 1, 2023, to March 30, 2024.

### Exposure Measures

Inflammation in this study was measured by CRP levels at ages 9, 15, and 17 years. We excluded measures of CRP levels greater than 1 mg/dL to minimize potential confounding by current infection or chronic inflammatory illness. Measures at birth were excluded due to high levels of missingness, and measures at age 24 years were excluded due to proximity to outcome data. The scores were standardized (*z* transformed) at each time point. We did not normalize the data because we expected the existence of latent classes within the data, and the process of normalizing the data can lose the variability vital for the delineation of latent classes.^20^ The eMethods in Supplement 1 show a sensitivity analysis using the log-transformed CRP levels. eTable 1 in Supplement 1 shows the output for the latent class growth analysis (LCGA), and eFigure 1 in Supplement 1 shows the class trajectories.

### Outcome Measures

#### Mental Health

We included outcomes of adult mental disorders at age 24 years^21^ to give sufficient temporal separation from the CRP measurement time points (eMethods in Supplement 1). Mental health outcomes at age 24 years included psychotic experiences (PEs), psychotic disorder, depression, and generalized anxiety disorder. Additionally, we included hypomanic symptoms, which were assessed at ages 22 to 23 years.

PEs were identified via the semistructured Psychosis-Like Symptom Interview.^22^ Assessment of PEs elicited if there were any of the 3 main positive psychotic symptoms, namely hallucinations, delusions, and thought interference, occurring in the last 6 months.^23^ Interviewers rated PEs as not present, suspected, or definitely present; for this study, cases of PEs were defined as definite PEs.^23^

Cases of psychotic disorder were not clinical diagnoses. They were defined as definite PEs that were not attributable to the effects of sleep or fever, occurred at least once per month during the previous 6 months, and (1) were very distressing, (2) negatively impacted social or occupational functioning, or (3) led to seeking help from a professional source.^23^

Depression was measured using a computerized version of the Clinical Interview Schedule–Revised.^24^ In this study, we examined all *International Statistical Classification of Diseases, 10th Revision*^25^ (*ICD-10*)–defined severities of depression to investigate not only the diagnosis but also the severity.

Generalized anxiety disorder was calculated using the Clinical Interview Schedule–Revised structured interview via *ICD-10* criteria.^24^

Hypomania symptoms were defined using the Hypomania Checklist–32. A score of 14 or higher out of 32 was considered hypomania.^26^

#### Cardiometabolic Health

We examined an age-appropriate measure of glucose-insulin sensitivity via the Homeostasis Model Assessment (HOMA2) score. This is a method for assessing beta cell function and IR.^27^

### Covariates

Covariates were chosen based on existing literature as likely to have an impact on childhood inflammation and/or adult mental health: biological sex at birth, self-reported race (White vs non-White [Bangladeshi, Black African, Black Caribbean, Other Black, Chinese, Indian, Pakistani, and other]; dichotomized due to the low levels of diversity within the cohort, which was 98.02% White and had very few or 0 recorded numbers in the remaining racial subgroups), preterm birth, body mass index (BMI; calculated as weight in kilograms divided by height in meters squared) recorded at the same time points as CRP levels, family adversity index, and parent-reported child health (ages 8 and 13 years) and emotional symptoms at age 9 years from the Strengths and Difficulties Questionnaire (SDQ). See the eMethods in Supplement 1 for further details on covariates. eTable 4 in Supplement 1 shows the levels of missingness and which variables were imputed.

### Statistical Analysis

We obtained descriptive and frequency statistics for our main variables of interest using SPSS version 29 statistical software (IBM Corp). We conducted LCGA on the CRP levels at ages 9, 15, and 17 years using Mplus version 8 software (Muthén & Muthén) to identify whether the population could be split into groups with differing levels of inflammation across childhood and adolescence. The differences between classes in rates of categorical variables were tested using χ^2^ tests. Differences in continuous variables were tested via analyses of variance. Finally, logistic and linear regression analyses were used in SPSS version 29 to investigate associations between the classes and mental and cardiometabolic outcomes in young adulthood. Two-tailed *P* < .05 was considered statistically significant.

#### LCGA

Classes were adjusted for BMI recorded at each time point. LCGA models were fitted iteratively by increasing the number of classes (2-6 classes).^28^ The LCGA model with the best fit was selected according to the Bayesian information criteria (BIC; lower values indicating better fit), Vuong-Lo-Mendell-Rubin (VLMR test; *P* < .05 for a K class model indicating a better fit than the K − 1 class model), and entropy (values closer to 1 indicating clear class delineation).^28^ We also selected the classification where the smallest class had a sample size greater than 1%. Missing values in LCGA were handled by the full information maximum likelihood estimation method.

#### Regression Analysis

For binary outcomes, we conducted logistic regression analyses for each outcome. The derived latent classes from the LCGA were used as the predictor, with the largest class (stable average levels of inflammation) used as the reference class. Odds ratios (ORs) and 95% CIs represent the increase in the risk of outcome per class compared with the reference class. Continuous outcomes were analyzed using linear regression analysis, where β coefficients represent the increase in outcome units per class compared with the reference class. For all outcomes, we conducted unadjusted and adjusted regression analyses. For all regression analyses, *P* values were corrected for the number of outcomes tested using the Holm-Bonferroni method to account for the risk of multiple testing bias. To help address the risk of missing-data bias, the analysis was weighted by conducting logistic regressions to identify factors significantly associated with attrition. The eMethods in Supplement 1 provide details of the inverse probability weighting. eTable 3 in Supplement 1 shows significant differences between participating and nonparticipating groups.

## Results

A total of 6556 participants (3303 [50.4%] female) were allocated to a class from the LCGA. Table 1 shows demographic comparisons of key variables for the entire cohort and between the 3 CRP classes. We found that higher CRP classes were proportionally more female, had higher BMI, and had significantly different CRP levels at each time point. eTable 2 in Supplement 1 shows the numbers of each outcome in each CRP class.

**Table 1.** Characteristics of CRP Level Trajectory Classes^a^

Characteristic	ALSPAC cohort (N = 15 645)	Reference class (n = 6109)	Early peak (n = 197)	Late peak (n = 250)	Statistic^b^	*P* value
Sex, No. (%)^c^						
Female	7348 (48.9)	3037 (46.8)	114 (58.2)	152 (60.8)	χ^2^_2_ = 16.48	<.001
Male	7691 (51.1)	3064 (50.2)	82 (41.8)	98 (39.2)		
Preterm births, No. (%)	856 (10.1)	288 (8.0)	8 (6.7)	12 (9.0)	χ^2^_2_ = 0.45	.80
Total family adversity index score, mean (SD)	4.38 (4.31)	3.89 (3.99)	4.43 (4.54)	4.35 (4.37)	*F*_25572_ = 2.65	.07
Race and ethnicity, No. (%)^d^						
White	12 062 (97.4)	5390 (98.0)	173 (99.4)	221 (97.4)	χ^2^_2_ = 2.29	.32
Non-White	326 (2.6)	100 (2.0)	<5 (<2.9)	6 (2.6)		
Rated as unhealthy, No. (%)^e^						
Age 8 y	122 (1.5)	51 (1.1)	7 (4.7)	<5 (<1.5)	χ^2^_2_ = 16.05	<.001
Age 13 y	108 (1.6)	50 (1.2)	<5 (<3.6)	<5 (<2.6)	χ^2^_2_ = 2.27	.03
CRP, mean (SD), mg/dL						
Age 9 y	0.056 (0.102)	0.039 (0.047)	0.479 (0.178)	0.067 (0.075)	*F*_25007_ = 5046.88	<.001
Age 15 y	0.085 (0.127)	0.070 (0.094)	0.199 (0.247)	0.294 (0.259)	*F*_23419_ = 372.59	<.001
Age 17 y	0.109 (0.142)	0.081 (0.080)	0.110 (0.119)	0.556 (0.163)	*F*_23202_ = 2569.20	<.001
BMI, mean (SD)						
Age 9 y	17.72 (2.91)	17.56 (2.71)	19.03 (4.01)	18.37 (3.02)	*F*_25900_ = 33.28	<.001
Age 15 y	21.46 (3.57)	21.34 (3.40)	22.59 (4.65)	22.52 (4.02)	*F*_24499_ = 19.44	<.001
Age 17 y	22.87 (4.22)	22.65 (3.94)	23.74 (4.78)	24.10 (4.84)	*F*_24142_ = 16.51	<.001
HOMA2 score at age 24 y, mean (SD)	1.26 (1.13)	1.24 (1.12)	1.45 (1.07)	1.30 (1.01)	*F*_22609_ = 1.41	.24

Abbreviations: ALSPAC, Avon Longitudinal Study of Parents and Children; BMI, body mass index (calculated as weight in kilograms divided by height in meters squared); CRP, C-reactive protein; HOMA2, Homeostasis Model Assessment.

SI conversion factor: To convert CRP to milligrams per liter, multiply by 10.

^a^ Where cell counts are less than 5, ALSPAC requests that a value of <5 be recorded instead of the exact value to protect confidentiality and ensure no one is possibly identifiable.

^b^ Differences in binary outcomes between classes were compared via Pearson χ^2^ test, and differences in continuous outcomes between classes were compared via analysis of variance.

^c^ A small number of participants (3.9%) were missing data on sex (consent withdrawn from mother or not known).

^d^ Data were self-reported. Non-White includes Bangladeshi, Black African, Black Caribbean, Other Black, Chinese, Indian, Pakistani, and other. The analysis was dichotomized due to the low levels of diversity within the cohort, which had very few or 0 recorded numbers in the racial subgroups other than White. A total of 3257 participants (20.8%) were missing data on race and ethnicity.

^e^ Child health was rated by the parents; they were asked in interview if, over the last 4 weeks, they would describe the health of the child as very healthy, healthy, sometimes quite ill, or almost always unwell. Responses were analyzed as a binary outcome of healthy (which included responses of very healthy and healthy) and unhealthy (which included responses of sometimes quite ill and almost always unwell).

### Latent Classes of Inflammation

Table 2 shows the values of log-likelihood (VLMR, BIC, and entropy) for all models assessed (2-6 classes). Only the 2-class and 3-class models had VLMR *P* < .05. The 3-class model (entropy = 0.93) and 2-class model (entropy = 0.98) had high entropy values, demonstrating good classification precision. The 3-class model presented lower BIC values, implying a better model fit over the 2-class model, and thus the 3-class model was selected as having the best overall model fit.

**Table 2.** Bayesian Information Criterion, Vuong-Lo-Mendell-Rubin Likelihood Test P Values, and Entropy for Classes 2 Through 6 of the C-Reactive Protein Level Score

Classes, No.	Bayesian information criterion	Vuong-Lo-Mendell-Rubin *P* value	Entropy
2	30 238.797	.003	0.980
3	28 014.523	<.001	0.927
4	23 669.657	.50	0.927
5	21 615.562	.28	0.930
6	21 642.806	.58	0.894

The 3 derived classes of inflammation are presented in the Figure, showing the mean *z* scores from the CRP values for each class at each time point, ages 9, 15, and 17 years. Class 1, hereafter referred to as the reference group, had persistently low CRP levels with stable average *z* scores and composed 93% of the cohort (n = 6109). Further, there were 2 divergent groups, both with persistently raised levels of CRP. Class 2 had persistently raised levels of CRP with a peak at age 9 years (hereafter referred to as the early peak group [EPG]) and composed 3% of the cohort (n = 197). Class 3 had persistently raised CRP levels with a peak at age 17 years (hereafter referred to as the late peak group [LPG]) and composed 4% of the cohort (n = 250). eFigure 2 in Supplement 1 shows the mean CRP values for each class at each time point. eFigures 3, 4, and 5 show the mean (*z*-transformed) CRP values for each individual in the reference group, EPG, and LPG, respectively.

**Figure.**
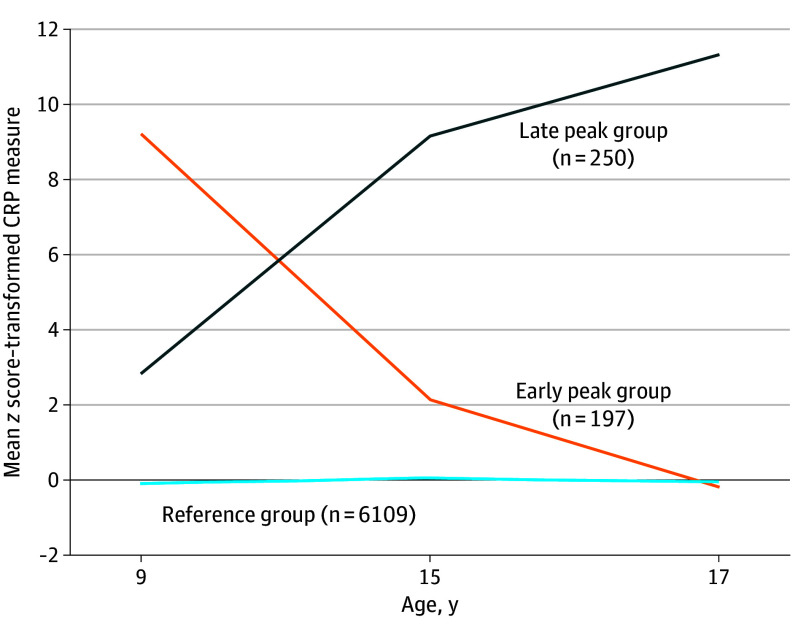
Latent Class Growth Analysis–Derived C-Reactive Protein (CRP) Level Trajectories CRP level trajectories form across childhood and adolescence. This graph depicts the *z* scores from the *Z*-transformed CRP values for each trajectory at each time point from the 3-class model, which had the best model fit. Class 1 represents the largest reference group with the least divergence from the mean with a stable average across time points. Class 2 represents the early peak group, whose CRP levels were persistently higher than those of the reference group but peaked early at age 9 years. Class 3 represents the late peak group, whose CRP levels were persistently higher than those of the reference group but peaked later at age 17 years.

### Associations Between Classes and Mental Health Outcomes

Weighted and adjusted logistic regression models showed that the EPG was associated with an increased risk of psychotic disorders at age 24 years (OR, 4.60; 95% CI, 1.81-11.70; *P* = .008) and PEs at age 24 years (OR, 2.30; 95% CI, 1.33-3.97; *P* = .02). Similarly, the EPG was associated with an increased risk of severe depression at age 24 years (OR, 4.37; 95% CI, 1.64-11.63, *P* = .02). Moderate depression at age 24 years was weakly associated since the association attenuated after correcting for multiple testing bias (OR, 1.97; 95% CI, 1.07-3.64; *P* = .10) (Table 3); thus, this result should be accepted with caution.

**Table 3.** Associations of Latent Classes of C-Reactive Protein Level Trajectory With Mental and Cardiometabolic Outcomes in Young Adulthood

Class	Unadjusted model			Adjusted model		
	OR (95% CI)	*P* value	Holm-Bonferroni method–corrected *P* value^a^	OR (95% CI)	*P* value	Holm-Bonferroni method–corrected *P* value^a^
Psychotic disorder at age 24 y						
Reference group, class 1	NA	NA	NA	NA	NA	NA
Early peak group, class 2	2.43 (1.04-5.68)^b^	.04^b^	.21	4.60 (1.81-11.70)^b^	.001^b^	.008^b^
Late peak group, class 3	0.67 (0.19-2.34)	.53	>.99	1.67 (0.46-6.04)	.44	>.99
Psychotic experiences at age 24 y						
Reference group, class 1	NA	NA	NA	NA	NA	NA
Early peak group, class 2	1.71 (1.14-2.56)^b^	.01^b^	.06	2.30 (1.33-3.97)^b^	.003^b^	.02^b^
Late peak group, class 3	0.95 (0.63-1.43)	.79	>.99	1.69 (1.06-2.69)^b^	.03^b^	>.99
Severe depression at age 24 y						
Reference group, class 1	NA	NA	NA	NA	NA	NA
Early peak group, class 2	5.05 (2.82-9.05)^b^	<.001^b^	.01^b^	4.37 (1.64-11.63)^b^	.003^b^	.02^b^
Late peak group, class 3	0.93 (0.34-2.54)	.89	>.99	2.06 (0.69-6.17)	.20	>.99
Moderate depression at age 24 y						
Reference group, class 1	NA	NA	NA	NA	NA	NA
Early peak group, class 2	1.91 (1.27-2.85)^b^	.002^b^	.02^b^	1.97 (1.07-3.64)^b^	.03^b^	.10
Late peak group, class 3	0.64 (0.39-1.07)	.09	.82	0.93 (0.50-1.73)	.81	>.99
Mild depression at age 24 y						
Reference group, class 1	NA	NA	NA	NA	NA	NA
Early peak group, class 2	1.45 (0.99-2.12)	.06	.22	1.38 (0.75-2.52)	.30	.56
Late peak group, class 3	0.70 (0.47-1.06)	.09	.82	1.09 (0.65-1.81)	.75	>.99
Hypomania at age 22-23 y						
Reference group, class 1	NA	NA	NA	NA	NA	NA
Early peak group, class 2	0.93 (0.68-1.27)	.64	>.99	1.31 (0.80-2.14)	.28	.56
Late peak group, class 3	0.94 (0.72-1.23)	.65	>.99	0.73 (0.48-1.13)	.16	>.99
Generalized anxiety disorder at age 24 y						
Reference group, class 1	NA	NA	NA	NA	NA	NA
Early peak group, class 2	1.39 (0.92-2.11)	.12	.37	1.56 (0.86-2.86)	.15	.45
Late peak group, class 3	1.16 (0.80-1.67)	.44	>.99	0.88 (0.49-1.56)	.66	>.99
HOMA2 score at age 24 y						
Reference group, class 1	NA	NA	NA	NA	NA	NA
Early peak group, class 2	0.05 (0.12-0.64)^b^^,^^c^	.004^b^	.03^b^	0.05 (0.01-0.62)^b^^,^^c^	.007^b^	.04^b^
Late peak group, class 3	0.001 (−0.18 to 0.19)^c^	.96	>.99	0.003 (−0.17 to 0.20)^c^	.85	>.99

Abbreviations: HOMA2, Homeostasis Model Assessment; NA, not applicable; OR, odds ratio.

^a^ *P* values corrected for multiple testing using the Holm-Bonferroni method.

^b^ Statistically significant value.

^c^ Data are presented as β (95% CI).

We did not find strong evidence for associations of the LPG with mental health outcomes (Table 3). Further, we did not find evidence for associations of any CRP class with outcomes of mild depression, hypomania, or generalized anxiety disorder (Table 3).

### Associations Between Classes and Cardiometabolic Outcomes

The adjusted linear regression analyses indicated that the EPG was associated with higher HOMA2 scores at age 24 years (β = 0.05; 95% CI, 0.01-0.62; *P* = .04) (Table 3). The LPG was not associated with the HOMA2 score at age 24 years.

## Discussion

We identified different classes of low-grade systemic inflammation across childhood and adolescence, with 2 groups with persistently increased inflammation—one with a peak in CRP level in midchildhood, and the other with a later peak in CRP level in late adolescence. Further, the EPG was associated with both mental (psychosis-spectrum disorder, depression) and cardiometabolic (HOMA2 score) outcomes in young adulthood. Our results support the hypothesis that having chronic low-grade inflammation in childhood and adolescence, peaking around midchildhood, may be a common risk factor for comorbid psychiatric and cardiometabolic disorders in adulthood.

First, and regarding inflammatory classes, we found a group with stable average levels of inflammation, which was the significant majority at more than 93% of the cohort. There were 2 divergent groups, both of which had CRP measures persistently over that of the reference group. The EPG (n = 197) had a mean CRP peak of approximately 0.5 mg/dL at age 9 years, which gradually decreased to just over 0.1 mg/dL at age 17 years. The implication is that this group of individuals was exposed to higher levels of inflammation earlier in life, which gradually resolved. The LPG (n = 250) started with a CRP measure of 0.066 mg/dL, which gradually increased to its peak of 0.55 mg/dL at age 17 years. This LPG had a steadily increasing CRP level, peaking later in adolescence. Other studies have also investigated CRP classes during childhood and/or adolescence.^9,29^ One study investigating CRP levels from ages 0 to 14 years found 2 latent classes, one with persistently low CRP levels throughout childhood (n = 430) and a second with persistently high and increasing CRP levels throughout childhood (n = 134), similar to our LPG.^29^ The second study^9^ looked at CRP classes from ALSPAC and found 4 classes. They had 3 similar class trajectories, termed low, similar to our reference group; increasing, similar to our LPG; and decreasing, similar to our EPG, along with a fourth class of persistently high.^9^ Both these studies and our study identified a majority group with persistently low CRP and divergent groups with periods of increased CRP, but with varying numbers of classes and patterns. Other research has uncovered possible causes of low-grade inflammation related to mental health, including genetics, maternal and infant infections, and early-life adversity.^30^ We have not examined this in this study, and further research is needed.

Second, we found significant associations between persistently increased inflammation in the EPG and psychotic disorder, PEs, severe depression, and increased HOMA2 score. Other studies looking at single time points have found longitudinal associations between childhood inflammation and psychosis-spectrum outcomes,^6^ depression,^6^ and IR^15^ but were unable to explore the role of persistent inflammation across childhood and adolescence. A previous study examined longitudinal CRP levels and associations with depression at age 18 years in ALSPAC participants.^9^ They found significant associations between a group with increasing CRP levels peaking at age 18 years and moderate or severe depression at age 18 years. This is different from our findings, with the group peaking at age 9 years and with decreasing CRP being associated with severe depression (along with psychotic disorder, PEs, and increased HOMA2 score) at age 24 years. The methods of the 2 studies differed in several key ways. First, Osimo et al^9^ only included people who had a full set of recordings of CRP level at each time point, giving a total sample size of 1561 participants. In contrast, our LGCA analysis uses data from a larger and more representative cohort of 6556 participants. Second, the previous study adjusted for BMI at age 18 years only, which may have limited the ability to capture the longitudinal impact of BMI on CRP levels across childhood. In our study, we adjusted for BMI at each individual time point during the LCGA; thus, we were better able to isolate the associations of CRP independent of BMI. Third, Osimo and colleagues log transformed their CRP values to help with normalization. This resulted in more homogeneously sized groups of potentially limited specificity; for example, their increasing CRP group composed 25% of their total population. In contrast, our classes resulted in an EPG consisting of just 3% of the total population.

Our findings are in keeping with the growing evidence of an association between inflammation and psychotic, depressive, and cardiometabolic disorders. Our hypothesis based on this new evidence is that persistently increased CRP reflects chronic low-grade inflammation that may peak at age 9 years and have downstream consequences in the central nervous system, such as microglia activation and subsequent alterations in brain structure and function,^23^ and in the periphery on glucose-insulin homeostasis at a critical developmental stage. This may ultimately lead to changes that significantly predispose to developing disorders such as psychosis and depression in young adulthood while simultaneously predisposing to commonly co-occurring cardiometabolic disorders. The LPG did not have any increases in outcomes tested. One explanation may be that CRP is a trait marker of illness, that is, one that plays an antecedent, possibly causal role in the pathophysiology,^31^ which is why the EPG is at increased risk. This is as opposed to a state marker, which reflects the status of clinical manifestations in patients^31^ and is the pattern of increasing to the point of illness that is seen in the LPG.

### Strengths and Limitations

This study has several strengths. First, the sample was large, population based, and analyzed by a longitudinal design. Second, using LCGA to create classes of inflammation provides a more reliable measure of underlying inflammation to better identify population subgroups and allows us to assess dynamic changes in CRP level. Third, the range of robustly measured outcomes tested was wide. Fourth, the exposure (inflammation at ages 9-17 years) and outcomes (at age 24 years) were temporally separated, which reduces the likelihood of reverse causality of the outcome, eg, psychosis contributing to or causing the increased inflammation. Finally, we also examined the association of the same inflammation classes with both mental and cardiometabolic outcomes, demonstrating that the same exposure is associated with both outcomes.

We do acknowledge some limitations. First, we could not assess trends of inflammation earlier than age 9 years; consequently, we were unaware of the level of inflammation before age 9 years. Second, as is common in long-term population cohort studies, attrition was high (75.2%). However, we used methods, such as weighting and full information maximum likelihood estimation, to ensure representativeness in our results. Third, we included multiple outcomes, which may have increased the risk of type II statistical error; however, we accounted for this by using the Holm-Bonferroni method to correct *P* values. Fourth, regarding the potential for reverse causality, we adjusted for emotional symptoms at age 9 years using the emotional symptoms subscale of the Strengths and Difficulties Questionnaire; however, due to missingness in this variable, we used kNN imputation. Further, there is no measure of psychotic symptoms at age 9 years or earlier in the ALSPAC dataset, so we cannot definitively rule out reverse causality for psychosis-spectrum outcomes. Yet, we believe the risk of this is low because the onset of psychotic disorder by midchildhood is rare.^32^ We were also unable to adjust for HOMA2 score at age 9 years, so we cannot rule out reverse causality. Fifth, there were potentially important covariates we were unable to adjust for, including smoking, and with all observational analyses, there is a risk of residual confounding. Sixth, we examined only IR as a cardiometabolic outcome; further research investigating other cardiometabolic outcomes would be of value. Also, this study was unable to probe potential biological mechanisms, so further research is needed to unpack the association between inflammation and both mental health and cardiometabolic outcomes.

## Conclusions

Our study found that specific classes of low-grade systemic inflammation across childhood and adolescence were associated with later onset of mental health disorders, particularly psychosis and depression, with early rather than later inflammation potentially holding importance. Further, the EPG was also at higher risk of developing certain cardiometabolic disorders, such as IR. This study adds new information about the chronicity and timing of inflammation predating illness and provides insight into the co-occurrence of related cardiometabolic disorders.

## Notes

Supplement 1. **eMethods.** Supplemental Methods **eTable 1.** Output From the LCGA for 2-6 Class Model **eFigure 1.** LCGA trajectories and Raw CRP Measure for the Normalized Sensitivity Analysis **eFigure 2.** LCGA Trajectories and Raw CRP Measure **eFigure 3.** Estimated Means and Observed Individual Values Graph for the Reference Group **eFigure 4.** Estimated Means and Observed Individual Values Graph for the Early Peak Group **eFigure 5.** Estimated Means and Observed Individual Values Graph for the Late Peak Group **eTable 2.** Numbers of Mental and Cardiometabolic Health Outcomes in Each CRP Class **eTable 3.** Differences in Socio-Demographic Variables Between Non-Participating and Participating Subjects in the Study **eTable 4.** Amount of Missing Data for Each Predictor or Confounder Variable
eReferences

Supplement 2. Data Sharing Statement
