# Supplementary material for: Trajectories of Inflammation in Youth and Risk of Mental and Cardiometabolic Disorders in Adulthood
Source: JAMA Psychiatry. 2024 Aug 21;81(11):1130–7. doi: 10.1001/jamapsychiatry.2024.2193 (PMC11339695; doi:10.1001/jamapsychiatry.2024.2193)
Supplement: Supplement 1. — eMethods. Supplemental Methods eTable 1. Output From the LCGA for 2-6 Class Model eFigure 1. LCGA trajectories and Raw CRP Measure for the Normalized Sensitivity Analysis eFigure 2. LCGA Trajectories and Raw CRP Measure eFigure 3. Estimated Means and Observed Individual Values Graph for the Reference Group eFigure 4. Estimated Means and Observed Individual Values Graph for the Early Peak Group eFigure 5. Estimated Means and Observed Individual Values Graph for the Late Peak Group eTable 2. Numbers of Mental and Cardiometabolic Health Outcomes in Each CRP Class eTable 3. Differences in Socio-Demographic Variables Between Non-Participating and Participating Subjects in the Study eTable 4. Amount of Missing Data for Each Predictor or Confounder Variable eReferences [file jamapsychiatry-e242193-s001.pdf]

Supplemental Online Content

Palmer ER, Morales-Muñoz I, Perry BI, et al. Trajectories of inflammation in youth and risk of mental and cardiometabolic disorders in adulthood. *JAMA Psychiatry*. Published online August 21, 2024. doi:10.1001/jamapsychiatry.2024.2193

eMethods. Supplemental Methods
eTable 1. Output From the LCGA for 2-6 Class Model
eFigure 1. LCGA Trajectories and Raw CRP Measure for the Normalized Sensitivity Analysis
eFigure 2. LCGA Trajectories and Raw CRP Measure
eFigure 3. Estimated Means and Observed Individual Values Graph for the Reference Group
eFigure 4. Estimated Means and Observed Individual Values Graph for the Early Peak Group
eFigure 5. Estimated Means and Observed Individual Values Graph for the Late Peak Group
eTable 2. Numbers of Mental and Cardiometabolic Health Outcomes in Each CRP Class
eTable 3. Differences in Socio-Demographic Variables Between Non-Participating and Participating Subjects in the Study
eTable 4. Amount of Missing Data for Each Predictor or Confounder Variable
eReferences

This supplemental material has been provided by the authors to give readers additional information about their work.

eMethods. Supplemental Methods

1. Further Details On The ALSPAC Data Set

Pregnant women resident in Avon, UK with expected dates of delivery between 1st April 1991 and 31st December 1992 were invited to take part in the study. 20,248 pregnancies have been identified as being eligible and the initial number of pregnancies enrolled was 14,541. Of the initial pregnancies, there was a total of 14,676 foetuses, resulting in 14,062 live births and 13,988 children who were alive at 1 year of age..(1) The cohort initially included 14,676 foetuses, resulting in 14,062 live births and 13,988 children who were alive at 1 year of age.. At age 7 years old, additional recruitment to bolster sampling was conducted with cases who had failed to join the study originally. The total sample size for analyses using any data collected after the age of seven is therefore 15,447 pregnancies, resulting in 15,658 foetuses. Of these14,901 children were alive at 1 year of age.(1) Initially, postal questionnaires regarding all aspects of child health and development were completed by parents from birth to age 7. Thereafter, the children attended an annual face-to-face assessment clinic which included assessments, interviews and physical tests.(1,2) Study data were collected and managed using REDCap electronic data capture tools hosted at the Universityof Bristol.(3) REDCap (Research Electronic Data Capture) is a secure, web-based software platform designed tosupport data capture for research studies. Please note that the studywebsite contains details of all the data that is available through a fully searchable data dictionary and variablesearch tool" and reference the following webpage: http://www.bristol.ac.uk/alspac/researchers/our-data/.

2. Further details on the Measure of inflammation

Samples were then immediately spun, frozen and stored at −80 °C. There was no evidence of freeze-thaw cycles during storage. High-sensitivity CRP (hs-CRP) was measured by automated particle-enhanced immunoturbidimetric assay by the same laboratory (Roche UK). All assay coefficients of variation were <5%. (4,5) Inflammation in this study was measured by CRP and recorded at birth (from a cord sample) at 9, 15, 17 and 24 years of age. These were collected from non-fasting blood samples. Of the CRP cord sample data, there were 4695 (30%) recorded values, however, 3828 of these (82% of recorded values) were recorded as 0. It was unclear whether this reflected a was a true 0, unmeasurable value or missing data. As a result, we excluded this time point from the LCGA. We also excluded data from the sample at age 24, due to proximity to outcome variables at age 24.
Scores over 10 were removed due to reflecting higher levels or acute stages of inflammation. At age 9: sixty of the 5105 recordings of CRP were >10, at age 15: sixty two of the 3513 recordings of CRP were> 10, and at age 17: seventy-nine of the 3311 recordings of CRP were >10. Two participants had CRP recordings >10 at two separate time points, and no one had CRP recordings of over 10 at all three time points. The missing values were handled in the LCGA in the same way any other missing values were, by full information maximization likelihood (FIML) estimation. FIML estimates parameters directly using all the information that is already contained in the incomplete data set under the "missing at random" assumption.(6) FIML has been demonstrated to produce unbiased estimates(7) and valid model-fit information.(8) At age 9: 6 of the 5105 recordings were over 10, at age 15: 62 of the 3513 recordings were over 10, and at age 17: 79 of the 3311 recordings were over 10. Of the whole cohort, only 2 people had CRP recordings of over 10 at two separate time points, and no one had CRP recordings of over 10 at all three time points.

3. Further details on Psychotic outcomes

PEs were established via semi-structured Psychosis-Like Symptom Interview (PLIKSi) conducted face-to-face by trained psychology graduates.(9) The coding of psychotic experiences followed definitions and the rating rules for the Schedules for Clinical Assessment in Neuropsychiatry Version 2.0.(10) The interrater reliability of the PLIKSi at age 24 was good, (Intraclass correlation: 0.81; 95% CI, 0.68–0.89, test-retest reliability: 0.9; 95% CI 0.83–0.95).(11) Assessment of PEs elicited if there were any of the three main positive psychotic symptoms namely hallucinations, delusions and thought interference, occurring in the last six months. Interviewers rated PEs as either not present, suspected, or definitely present, for this study, cases of PE's were defined as induvial assessed as having definite PEs.(4)

© 2024 Palmer ER et al. *JAMA Psychiatry.*

4. Further details on Depressive outcomes

Depression was measured using a computerised version of the Clinical Interview Schedule–Revised (CIS-R). The CIS-R is a widely used standardised self-administered tool for measuring depression aimed specially at community samples.(12) The CIS-R assesses symptoms of depression over the past 7 days and provides a diagnosis of either a mild, moderate or severe depressive episode based on the International Statistical Classification of Diseases, 10th Revision (ICD-10).(13) In this study we examined all severities of depression, to investigate not only the diagnosis, but also the severity of diagnosis.
The CIS-R assesses symptoms of depression over the past 7 days and provides a diagnosis of either a mild, moderate or severe depressive episode based on the International Statistical Classification of Diseases, 10th Revision (ICD-10). To be given a diagnosis of each severity, you need to have surpassed a certain score, so those who reach the threshold for a diagnosis of severe depression would have also surpassed the threshold for a diagnosis of Mild and Moderate depression, and will have a data label as Mild depression, moderate depression and severe depression.

5. Further details on Hypomania outcome

Hypomania symptoms were defined using the Hypomania Checklist, a self-report measure of lifetime experience of manic symptoms. Participants were asked to consider a time when they were in a "high or hyper" state and to endorse a number of statements about their emotions, thoughts, and behaviors at that time. We defined lifetime history of hypomania as follows: a score of 14 or more out of 32 hypomanic features; plus at least one response of either "negative consequences" or "negative plus positive consequences"; plus a report that these mood changes caused a reaction in others; plus a duration of "2–3 days" or more. To note, the Hypomania Checklist was only used when the young person was 22-23 years, and not at 24 years old. Therefore, this was the only mental health outcome which was collected at 22-23 years, instead of at 24 years.

6. Further details on Generalised anxiety disorder

These outcomes were also calculated based on the the CIS-R structured interview, the same used to determine depressive disorder. It covers 14 types of common mental disorder symptoms (somatic symptoms, fatigue, concentration and forgetfulness, depression, depressive ideas, worry, anxiety, sleep problems, irritability, worry about physical health, phobias, panic, compulsions and obsessions), and six (non-mutually exclusive) ICD-10 disorders (Generalized anxiety disorder, depression, phobias, obsessive-compulsive disorder, panic disorder, and common mental disorder not otherwise specified [NOS]), together with a continuous scale that reflects the overall severity of common mental disorder psychopathology. The CIS-R has been shown to be equally reliable when administered by interviewer or in a computer-assisted self-administered format. It has been widely used in population surveys.

7. Further details on HOMA2 score

The homeostasis model assessment (HOMA) score is a method for assessing beta-cell function and insulin resistance (IR) from basal (fasting) glucose and insulin and is a way of estimating insulin resistance.(14) The HOMA2 score is an updated way of calculating this score.(15) This was done by entering the recorded fasting glucose and fasting insulin recording from age 24 into a HOMA2 calculator available here (https://www.rdm.ox.ac.uk/about/our-clinical-facilities-and-mrc-units/DTU/software/homa) form the Radcliffe department of medicine, University of Oxford.

8. Further details on covariates

In this analysis, we adjusted for variables that we felt were likely to have a significant impact on either rates of mental health disorder at age 24 and/or childhood and adolescent inflammation. Accordingly, we adjusted for sex, as there are known sex differences in rates of many mental health conditions such as anxiety, depression (16) and psychosis. (17) For this variable we used the sex label given in the data set, which was either male or female. Ethnicity, due to low levels of diversity in the cohort, that is, low level of non-white ethnicity labels, we treated ethnicity as a binary outcome of white and non-white. We also adjusted for Preterm birth, which has been associated with poor mental health and educational outcomes.(18) Body mass index (BMI) (weight (kg)/height (m2)) was

measured at various time points, and was included as it is known to impact CRP, with higher BMI being associated with high CRP. (19) We adjusted for BMI during the LCGA, as it meant we could adjust for BMI at each time point that the CRP was recorded. Due to the level of missingness in this data, we used the *k*-nearest neighbours (Knn) imputation algorithm of the VIM package in R (20) (using recommended settings) to replace missing data. The Knn algorithm is sensitive and robust to different data types, and performs comparatively well to other methods of imputation such as multiple imputation using chained equations.(21) Family adversity index (FAI), is a measure of childhood adversity and socioeconomic status and is measured during pregnancy (long index), at 2 years (long index), and at 4 years (short index). The long index version is comprised of 18 items (long index) assessing 10 factors relevant to family adversity, including mothers' age, housing, parental education, household finances, parents' relationship, family size, social network, parental criminal activity and maternal psychopathology.(22) The short index version excludes social, practical, and financial support. Each of the items is listed as either present, given a score of 1, or absence, and given 0. We used a total FAI, which was the total scores from each time point (pregnancy, age 2 and age) summed together. FAI was included as a confounder as early adversity is a well-established risk factor for poor mental health.(23)

Child health was rated by the parents; they were asked in interview if, over the last 4 weeks, they would describe the health of the child as very healthy, healthy, sometimes quite ill, almost always unwell. We created a binary outcome of healthy (which included responses of very healthy and healthy) and unhealthy (which included responses of sometimes quite ill and almost always unwell). We adjusted for this as a recent illness may have had an altered CRP as an inflammatory marker. It was selected at ages 8 and 13 as these were closest to the CRP time measures.

Childhood emotional problems were assessed at age 9 via the Strength and Difficulties Questionnaire – emotional symptoms subscore.(24) The SDQ screens for emotional symptoms, hyperactivity/inattention and peer relationship problems. We used the emotional symptoms subscore as an adjustment variable to account for any individuals who may have already had some emotional or mental health problems before the first measure of CRP. Due to a large reduction in the available sample size when the SDQ was included as an adjustment variable to regression models testing associations of trajectory class membership with outcomes, we used Knn imputation to replace missing values as we did for BMI.

9. Further details on Latent Class Growth Analysis

The LCGA was conducted using Mplus v8. CRP scores at ages 9, 15 and 17 were used. BMI was controlled for at each time point, using the BMI measured at the same age as the CRP. Missing BMI scores were imputed using k-nearest neighbour single-imputation (KNN imputation) in R. Several models were fitted by increasing the number of classes.(25) The classification model with the best fit was selected according to specific fit indices namely Bayesian Information Criteria [BIC] and Vuong-Lo-Mendell-Rubin [VLMR] test.(25) Lower BIC values suggest a better model fit, while a significant VLMR value implies that a K-class model fits the data better than a (K-1) class model. We also used Entropy to select the best model fit; entropy with values approaching 1 indicates a clear delineation of the different classes. Missing values due to attrition were handled by the Full Information Maximum Likelihood estimation method. The derived latent classes that illustrate the different trajectories of CRP throughout childhood and adolescence were saved and imported to SPSS for use in the regression analyses.(4,25)

10. Sensitivity Analysis for Log transformed CRP Classes

As part of a sensitivity analysis, we also conducted the LCGA on Log transformed CRP data. For this, we removed any scores for crp recorded over 10, we then log10 transformed the CRP scores to normalise the data, and then Z transformed the data to standardise the data. We did the same for the imputed BMI scores to allow for adjusting for BMI at each time point as we did in the main analysis. We then re-ran the LCGA.

© 2024 Palmer ER et al. *JAMA Psychiatry.*

Class results

eTable 1. Output From the LCGA for 2-6 Class Model

Bayesian Information Criterion, Vuong-Lo-Mendell-Rubin Likelihood Test P Values and Entropy for Classes 2-6 of the Log Transformed CRP score

No. of Classes	BIC	VLMR-p Value	Entropy
2 Classes	30794.083	<0.001	0.662
3 Classes	30489.614	<0.001	0.566
4 Classes	30479.077	0.2675	0.567
5 Classes	30448.097	0.3215	0.591
6 Classes	30423.190	0.4442	0.632

BIC, Bayesian information criterion; VLMR, Vuong-Lo-Mendell-Rubin.

The output of the LCGA for 2-6 classes shows that only the 2 and 3-class models were significant. The 3-class model has a low BIC number, indicating better model fit and so was selected as the best class model. We did, however, notice the very low Entropy Values for the classes. Entropy, which is a reflection of class delineation, should be approaching 1; although there is no hard cut-off, entropy values of <0.8 imply poor class delineation.

Class Numbers and Proportions

- Class 1: n=4752 (72.5%)
- Class 2: n=342 (5.2%)
- Class 3: n=1461 (22.3%)

© 2024 Palmer ER et al. *JAMA Psychiatry.*

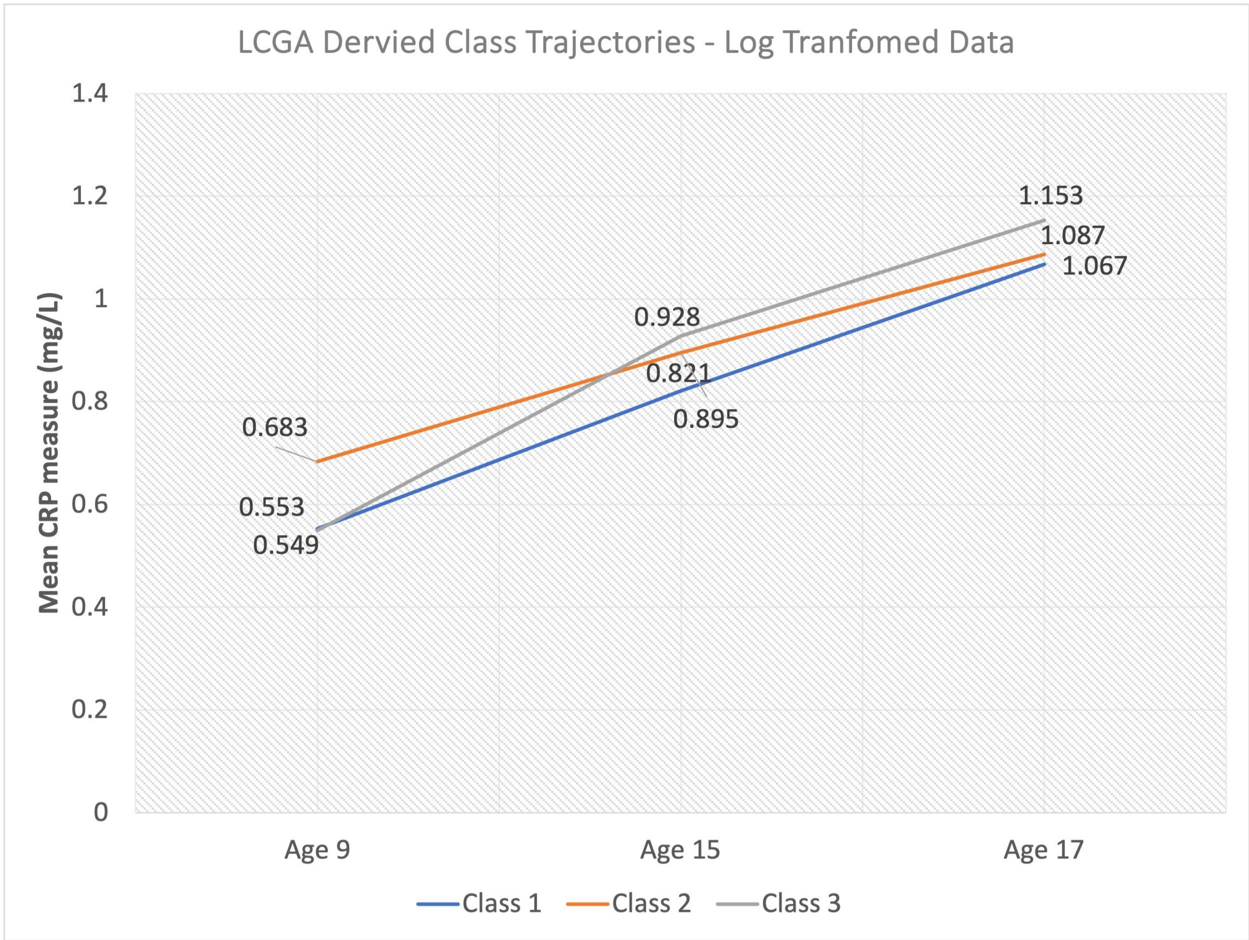

eFigure 1. LCGA Trajectories and Raw CRP Measure for the Normalized Sensitivity Analysis

CRP trajectories form across childhood and adolescence. This graph depicts the mean CRP result at each time point for each of the trajectories (classes), from the 3-class model that had the best model fit. Class 1 (the blue line) represent the largest class, with CRP generally the lowest, but slowly rising over the three time points. Class 2 (the orange line) represents a group who also had CRP rising over the three time point, and had the highest of all recordings of any of the groups at age 17. Finally, Class 3 (the green line) represents a group who had also had rising CRP over the three time points, but had CRP persistently over that of the reference group, but that peaked later at age 17.

You can see from this figure, as compared to eFigure 1, that the CRP trajectories of the three classes are very homologous. The overall trajectories and pattern are the same between them, increasing over the three time points. When comparing the CRP between classes at each time point, Via an ANOVA test, there were no significant differences between the CRP values at any time point (Age 9 [$F(2,5009) = 1.983$, $P=0.138$], Age 15 [$F(2,3418) = 2.225$, $P=0.108$], Age 17 [$F(2,320) = 1.027$, $P=0.358$]). Again, unlike in the main analysis Classes, where there was a significant difference in CRP between the classes at each time point (see Table 1).

Differences between main analysis classes and sensitivity analysis classes

By conducting the LCGA on the log-transformed (normalised) data it has derived 3 very homologous classes. This was to be expected with such low entropy values, implying poor class delineation. We believe that by normalising the data, it has "normalised out" the signal from the latent classes.

© 2024 Palmer ER et al. *JAMA Psychiatry.*

11. Details of how missing data was handled

To deal with missing data due to attrition, we conducted logistic regressions to identify significant factors associated with attrition. The individuals associated with attrition at 24 years old were more often boys, their mothers were younger when baby was born, the gestational age was shorter, they weighted less at birth, and they had higher socioeconomic levels.(4) Using variables associated with selective dropout as factors, we fitted a logistic regression model to determine weights for each individual using the inverse probability of response. The regression coefficients from this model were used to determine probability weights for the covariates in the main analyses.(4) Please see eTable 3 for details.

Supplementary Tables and Figures

12. eFigure 2. LCGA Trajectories and Raw CRP Measure

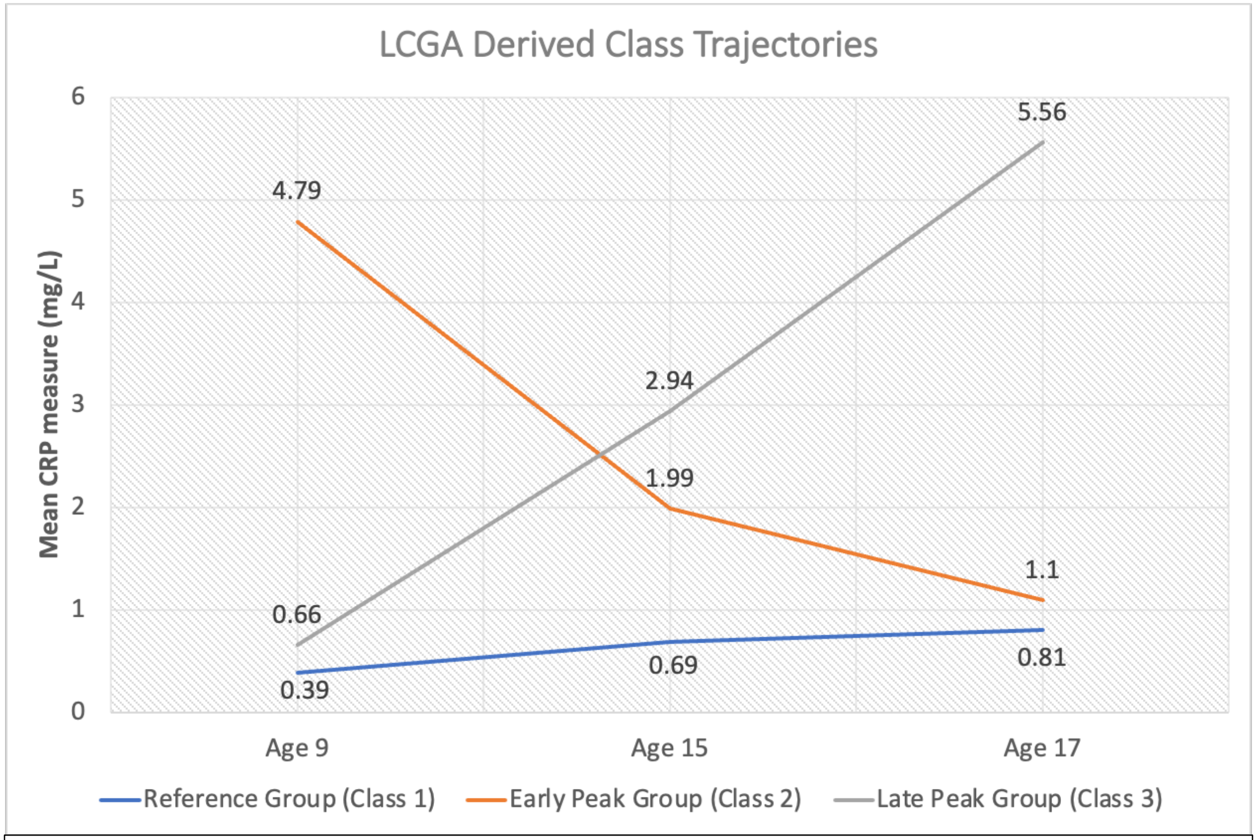

eFigure 2. CRP trajectories form across childhood and adolescence. This graph depicts the mean CRP result at each time point for each of the trajectories (classes), from the 3-class model that had the best model fit. Class 1 (the blue line) represent the largest reference group with persistently low CRP. Class 2 (the grey line) represents the early peak group who had CRP persistently over that of the reference group, but that peaked early at age 9. Finally, Class 3 (the orange line) represents the late peak group who had CRP persistently over that of the reference group, but that peaked later at age 17.

© 2024 Palmer ER et al. *JAMA Psychiatry.*

13. eFigure 3. Estimated Means and Observed Individual Values Graph for the Reference Group

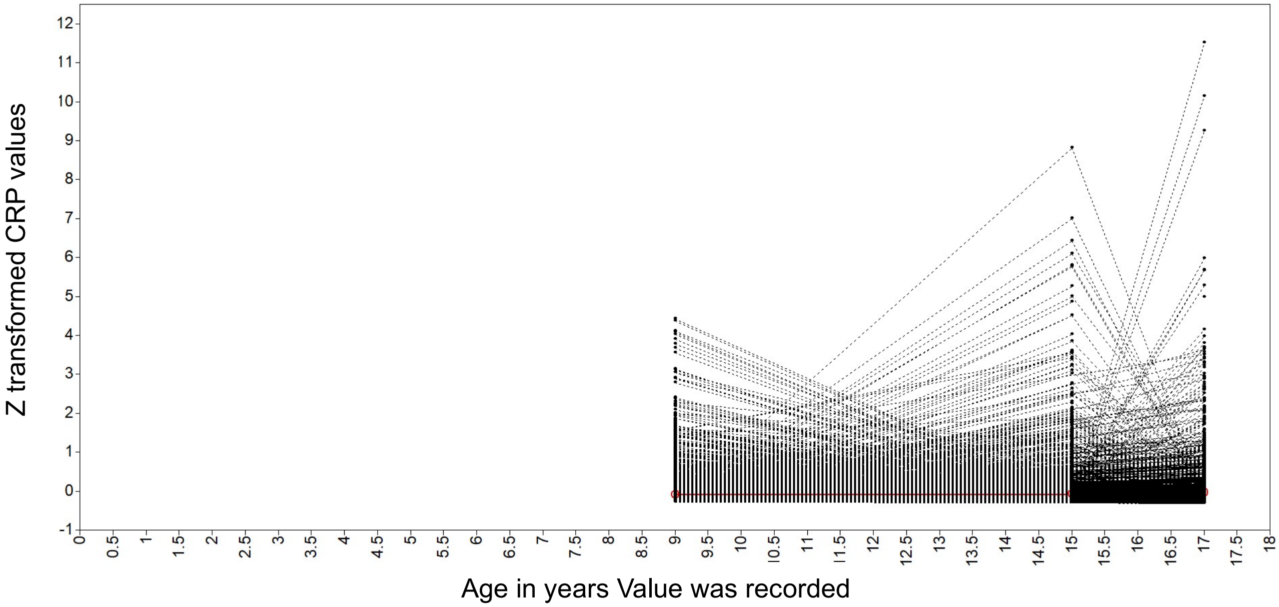

eFigure 3. Estimated means and observed individual values graph. This graph depicts the indivudal z transformed CRP values for each participant in class 1 (reference group) and the estimated mean values for the class in red.

14. eFigure 4. Estimated Means and Observed Individual Values Graph for the Early Peak Group

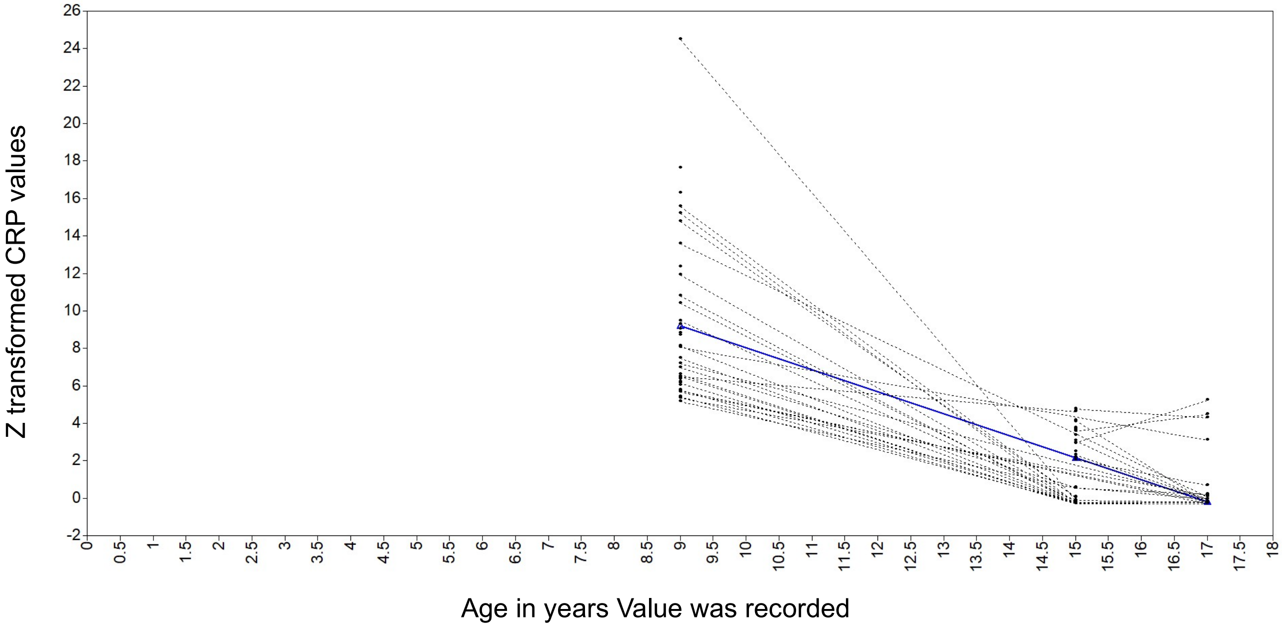

eFigure 4. Estimated means and observed individual values graph. This graph depicts the individual z transformed CRP values for each participant in class 2 (Early Peak group) and the estimated mean values for the class in Blue.

© 2024 Palmer ER et al. *JAMA Psychiatry.*

15. eFigure 5. Estimated Means and Observed Individual Values Graph for the Late Peak Group

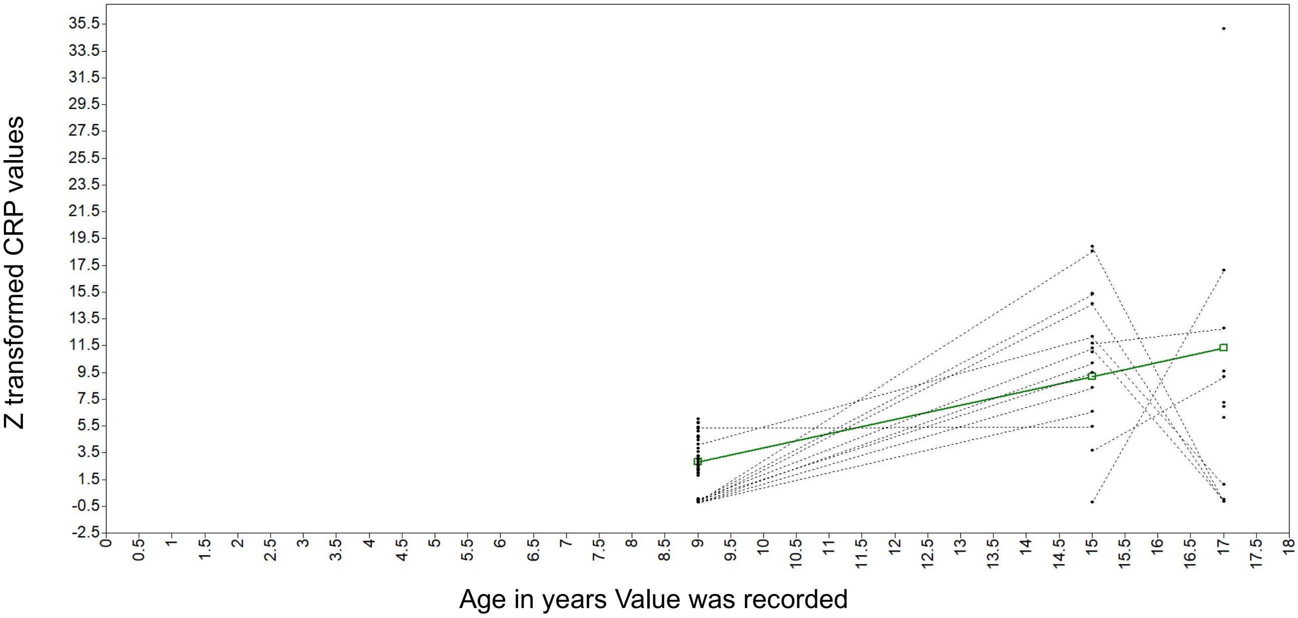

eFigure 5. Estimated means and observed individual values graph. This graph depicts the individual z transformed CRP values for each participant in class 3 (Late Peak group) and the estimated mean values for the class in green.

16. eTable 2. Numbers of Mental and Cardiometabolic Health Outcomes in Each CRP Class

	**Reference Group Class 1**	**Early Peak Group Class 2**	**Late Peak Group Class 3**
Psychotic Disorder	33 (1.21%)	<5[a] (<5.95%)	<5[a] (<3.91%)
Psychotic Expirences	208 (7.60%)	10 (11.90%)	8 (6.25%)
Severe Depression	35 (1.25%)	<5[a] (<5.88%)	<5[a] (<3.85%)
Moderate Depression	200 (7.17%)	10(11.76%)	6(4.62%)
Mild depression (%)	287 (10.28%)	12(14.12%)	9(6.92%)
Severe Depression and Psychosis Disorder	5 (0.19%)	<5[a,b] (<5.95%)	<5[a,b] (<3.94%)
Hypomania	1320 (64.45%)	39 (63.93%)	60 (65.22%)
Generlised Anxiety Disorder	255 (9.14%)	10 (11.76%)	12 (3.30%)
Diabetes	11 (0.40%)	<5[a,b] (<5.75%)	<5[a] (<3.94%)
Insulin resistance	449 (18.49%)	21 (30.00%)	21 (18.92%)

a – Where cell counts are less than 5 (and including zero), ALSPAC requests that <5 be recorded instead of the exact value in order to protect confidentiality.
b – Where the exact value may include zero.

© 2024 Palmer ER et al. *JAMA Psychiatry.*

17. **eTable 3. Differences in Socio-Demographic Variables Between Non-Participating and Participating Subjects in the Study**

	Non-participating group in the study		Participating group in the study (at 24 years old)		Non-participating versus participating	
	Mean	*SD*	*Mean*	*SD*	*OR (95% CI)*	*p*
Maternal age when born	27.49	4.99	29.45	4.56	1.08 (1.07, 1.09)	<0.001
Gestational age	38.00	6.22	39.49	1.80	1.09 (1.08, 1.11)	<0.001
Birth weight, kg	3371.23	596.18	3410.33	532.74	1.25 (1.05, 1.20)	<0.001
Family Adversity score	4.74	4.47	3.61	3.84	0.94 (0.93, 0.95)	<0.001
	**Non-participating group in the study**		**Participating group in the study**			
	N	*%*	*N*	*%*		
Sex						
Male / Female	6233 / 4919	55.9 / 44.1	1458 / 2429	37.5 / 62.5	0.47 (0.44, 0.51)	<0.001
Ethnicity						
White / Other	8657 / 248	97.2 / 2.8	3405 / 78	97.8 / 2.2	1.25 (0.97, 1.62)	0.089

The individuals associated with attrition at 24 years were more often boys, their mothers were younger when the baby was born, the gestational age was shorter, they weighed less at birth, and they had higher socioeconomic levels.

© 2024 Palmer ER et al. *JAMA Psychiatry.*

18. eTable 4. Amount of Missing Data for Each Predictor or Confounder Variable

Variable	Number (percentage) Missing Participants with Depression assessment (3966)
CRP age 9	1704 (42.8%)
CRP age 15	1972 (49.5%)
CRP age 17	1929 (48.6%)
CRP class allocation	960 (24.2)
BMI age 9*	688 (17.3%)
BMI age 15*	1002 (25.3%)
BMI age 17*	924 (23.3%)
Sex assigned at birth	<5[a] (<0.1%)
Ethnicity	409 (10.3%)
Preterm Birth	1662 (41.1%)
Strengths and difficulties (SDQ)* (emotional symptoms sub score)	753 (19.0%)
Child Health age 8	853 (21.5%)
Child Health age 13	997 (25.1%)
Family adversity Index	603 (15.2%)

CRP, C-reactive protein; BMI, Body Mass Index; HOMA2, homeostasis model assessment
*Variables with an asterisk were imputed using Knn imputation for use as a covariate
a – Where cell counts are less than 5, ALSPAC requests that <5 be recorded instead of the exact value in order to protect confidentiality.

© 2024 Palmer ER et al. *JAMA Psychiatry.*

19. eReferences

1. Boyd A, Golding J, Macleod J, Lawlor DA, Fraser A, Henderson J, et al. Cohort Profile: The 'Children of the 90s'—the index offspring of the Avon Longitudinal Study of Parents and Children. International Journal of Epidemiology. 2013 Feb;42(1):111–27.

2. Fraser A, Macdonald-Wallis C, Tilling K, Boyd A, Golding J, Davey Smith G, et al. Cohort Profile: The Avon Longitudinal Study of Parents and Children: ALSPAC mothers cohort. International Journal of Epidemiology. 2013 Feb 1;42(1):97–110.

3. Harris PA, Taylor R, Thielke R, Payne J, Gonzalez N, Conde JG. Research electronic data capture (REDCap)--a metadata-driven methodology and workflow process for providing translational research informatics support. J Biomed Inform. 2009 Apr;42(2):377–81.

4. Morales-Muñoz I, Palmer ER, Marwaha S, Mallikarjun PK, Upthegrove R. Persistent Childhood and Adolescent Anxiety and Risk for Psychosis: A Longitudinal Birth Cohort Study. Biological Psychiatry. 2022 Aug;92(4):275–82.

5. Perry BI, Zammit S, Jones PB, Khandaker GM. Childhood inflammatory markers and risks for psychosis and depression at age 24: Examination of temporality and specificity of association in a population-based prospective birth cohort. Schizophrenia Research. 2021 Apr;230:69–76.

6. Kline RB. Principles and Practice of Structural Equation Modeling. Guilford Publications; 2023. 514 p.

7. The Relative Performance of Full Information Maximum Likelihood Estimation for Missing Data in Structural Equation Models: Structural Equation Modeling: A Multidisciplinary Journal: Vol 8, No 3 [Internet]. [cited 2024 Apr 24]. Available from: https://www.tandfonline.com/doi/abs/10.1207/S15328007SEM0803_5

8. Enders CK. A Primer on maximum likelihood algorithms available for use with missing data. Structural Equation Modeling. 2001;8(1):128–41.

9. Zammit S, Kounali D, Cannon M, David AS, Gunnell D, Heron J, et al. Psychotic Experiences and Psychotic Disorders at Age 18 in Relation to Psychotic Experiences at Age 12 in a Longitudinal Population-Based Cohort Study. AJP. 2013 Jul;170(7):742–50.

10. Wing JK. SCAN: Schedules fonr Clinical Assessment in Neuropsychiatry. Arch Gen Psychiatry. 1990 Jun 1;47(6):589.

11. Sullivan SA, Kounali D, Cannon M, David AS, Fletcher PC, Holmans P, et al. A Population-Based Cohort Study Examining the Incidence and Impact of Psychotic Experiences From Childhood to Adulthood, and Prediction of Psychotic Disorder. AJP. 2020 Apr 1;177(4):308–17.

12. Lewis G, Pelosi AJ, Araya R, Dunn G. Measuring psychiatric disorder in the community: a standardized assessment for use by lay interviewers. Psychol Med. 1992 May;22(2):465–86.

13. Perry BI, Stochl J, Upthegrove R, Zammit S, Wareham N, Langenberg C, et al. Longitudinal Trends in Childhood Insulin Levels and Body Mass Index and Associations With Risks of Psychosis and Depression in Young Adults. JAMA Psychiatry. 2021 Apr 1;78(4):416.

© 2024 Palmer ER et al. *JAMA Psychiatry.*

14. Wallace TM, Levy JC, Matthews DR. Use and Abuse of HOMA Modeling. Diabetes Care. 2004 Jun 1;27(6):1487–95.

15. Levy JC, Matthews DR, Hermans MP. Correct Homeostasis Model Assessment (HOMA) Evaluation Uses the Computer Program. Diabetes Care. 1998 Dec 1;21(12):2191–2.

16. Altemus M, Sarvaiya N, Neill Epperson C. Sex differences in anxiety and depression clinical perspectives. Frontiers in Neuroendocrinology. 2014 Aug 1;35(3):320–30.

17. Ochoa S, Usall J, Cobo J, Labad X, Kulkarni J. Gender Differences in Schizophrenia and First-Episode Psychosis: A Comprehensive Literature Review. Schizophr Res Treatment. 2012;2012:916198.

18. O'Nions E, Wolke D, Johnson S, Kennedy E. Preterm birth: Educational and mental health outcomes. Clin Child Psychol Psychiatry. 2021 Jul;26(3):750–9.

19. Nappo A, Iacoviello L, Fraterman A, Gonzalez-Gil EM, Hadjigeorgiou C, Marild S, et al. High-sensitivity C-reactive Protein is a Predictive Factor of Adiposity in Children: Results of the Identification and prevention of Dietary- and lifestyle-induced health Effects in Children and InfantS (IDEFICS) Study. Journal of the American Heart Association. 2(3):e000101.

20. Kowarik A, Templ M. Imputation with the R Package VIM. Journal of Statistical Software. 2016 Oct 20;74:1–16.

21. Mandel J SP. A Comparison of Six Methods for Missing Data Imputation. J Biom Biostat [Internet]. 2015 [cited 2024 Apr 23];06(01). Available from: https://www.omicsonline.org/open-access/a-comparison-of-six-methods-for-missing-data-imputation-2155-6180-1000224.php?aid=54590

22. Collin SM, Tilling K, Joinson C, Rimes KA, Pearson RM, Hughes RA, et al. Maternal and Childhood Psychological Factors Predict Chronic Disabling Fatigue at Age 13 Years. Journal of Adolescent Health. 2015 Feb 1;56(2):181–7.

23. Wadman R, Hiller RM, Clair MCS. The influence of early familial adversity on adolescent risk behaviors and mental health: Stability and transition in family adversity profiles in a cohort sample. Development and Psychopathology. 2020 May;32(2):437–54.

24. Goodman R. Psychometric properties of the strengths and difficulties questionnaire. J Am Acad Child Adolesc Psychiatry. 2001 Nov;40(11):1337–45.

25. Jung T, Wickrama KAS. An Introduction to Latent Class Growth Analysis and Growth Mixture Modeling. Social & Personality Psych. 2008 Jan;2(1):302–17.

© 2024 Palmer ER et al. *JAMA Psychiatry.*
