# Supplementary material for: Trajectories of Inflammation in Youth and Risk of Mental and Cardiometabolic Disorders in Adulthood
Source: JAMA Psychiatry. 2024 Aug 21;81(11):1130–7. doi: 10.1001/jamapsychiatry.2024.2193 (PMC11339695; doi:10.1001/jamapsychiatry.2024.2193)
Supplement: Supplement 2. — Data Sharing Statement [file jamapsychiatry-e242193-s002.pdf]

Data Sharing Statement

Palmer. Trajectories of Inflammation in Youth and Risk of Mental and Cardiometabolic Disorders in Adulthood. *JAMA Psychiatry*. Published August 21, 2024.
doi:10.1001/jamapsychiatry.2024.2193

Data
Data available: No

Additional Information
Explanation for why data not available: The data in this study was from the large UK-based Avon Longitudinal Study of Parents and Children (ALSPAC) cohort study, the data is available from them. Any derived variables can be shared by contacting the corresponding author.
